# Cardiac Assist Devices: Early Concepts, Current Technologies, and Future Innovations

**DOI:** 10.3390/bioengineering6010018

**Published:** 2019-02-15

**Authors:** Jooli Han, Dennis R. Trumble

**Affiliations:** Department of Biomedical Engineering, Carnegie Mellon University, Pittsburgh, PA 15213, USA; dtrumble@andrew.cmu.edu

**Keywords:** cardiac assist devices, congestive heart failure, LVAD, destination therapy

## Abstract

Congestive heart failure (CHF) is a debilitating condition that afflicts tens of millions of people worldwide and is responsible for more deaths each year than all cancers combined. Because donor hearts for transplantation are in short supply, a safe and durable means of mechanical circulatory support could extend the lives and reduce the suffering of millions. But while the profusion of blood pumps available to clinicians in 2019 tend to work extremely well in the short term (hours to weeks/months), every long-term cardiac assist device on the market today is limited by the same two problems: infections caused by percutaneous drivelines and thrombotic events associated with the use of blood-contacting surfaces. A fundamental change in device design is needed to address both these problems and ultimately make a device that can support the heart indefinitely. Toward that end, several groups are currently developing devices without blood-contacting surfaces and/or extracorporeal power sources with the aim of providing a safe, tether-free means to support the failing heart over extended periods of time.

## 1. The Need for Mechanical Circulatory Support

Congestive heart failure (CHF) is a progressive condition in which cardiac function deteriorates over time. It is most common among people 65 years or older, but practically anyone can be at risk as the causes of heart failure include everything from coronary artery disease, high blood pressure, and congenital heart defects to myocarditis, abnormal heart rhythms, valve disease, diabetes, and obesity. The most common symptoms of the disease include shortness of breath and fatigue, and it is often diagnosed via blood tests, electrocardiograms, echocardiograms, stress tests, coronary angiograms, and chest x-rays [1] CHF remains one of the most costly diseases in the industrialized world, both in terms of healthcare dollars and the loss of human life. It is estimated that 26 million people currently suffer from CHF worldwide, including 5.8 million people in the United States where the economic impact exceeds $40 billion per year in medical costs and lost productivity. Worse still, roughly half of all people who develop CHF die within five years of diagnosis due to the limitations of current long-term treatment strategies [2,3]. Cardiac transplantation is generally considered to be the best recourse for end-stage CHF patients, but this treatment option is not available to most patients as the number of donated hearts is restricted by roughly 2200 hearts per year in the U.S. [4]. Pharmacologic therapies can improve heart function in the short term and relieve the symptoms associated with CHF, but are unable to restore and maintain normal heart function over the long term [5,6]. Therefore, decades of development work have focused on cardiac assist devices (CADs) as an alternate solution for end-stage CHF patients. 

### 1.1. Bridge Devices

CADs are often categorized according to duration of support. If a device lasts from hours to weeks as a means to stabilize patients until longer-term mechanical support can be implemented, it is considered to be a ‘bridge-to-device (BTD)’ [7]. BTDs were commonly used for myocardial recovery and mitral valve replacement from the 1970s through early 1980s. Nowadays, only about 25% of cases use this temporary treatment option, typically for one to four weeks post-operation [8]. 

CADs may also be used to provide circulatory support to patients on the waiting list for heart transplantation, in which case they are considered to be ‘bridge-to-transplant (BTT)’ devices. While BTT devices do not last longer than 2 years on average, the longevity of CADs is much better today compared to that of the mid-80s where the typical working life of these devices was only about a month [9]. Yet, this improvement does not increase the total number of patients who can receive a transplanted heart, but rather increases the chances of receiving a transplanted heart for patients receiving CAD treatment while proportionally reducing the odds for those who do not [9,10]. 

In rare instances that are difficult to predict, some patients recover cardiac function while under CAD support and no longer need heart transplantation. These cases are categorized—more often in retrospect than by design—as ‘bridge-to-recovery (BTR)’ [11]. 

### 1.2. Destination Therapy

Currently, the most ambitious unmet goal in the CAD field is to develop a cardiac support system for long-term or permanent use. A safe, reliable, durable, implantable support mechanism leading to the preservation, or even restoration, of cardiac competence and coronary flow that completely frees patients from the need for heart transplantation would be considered an effective device for ‘destination therapy (DT)’ [12]. In order to achieve this goal, researches have focused on overcoming several major failure modes associated with extended circulatory support. In this paper, we review the historical efforts, contemporary technologies, and up-to-date cutting-edge innovations that have been made to develop durable and reliable devices that both support cardiac function for long-term survival and also provide for better patient quality-of-life. 

## 2. The History of Cardiac Assist Devices

### 2.1. The Beginning

The concept of artificial blood pumps can be traced as far back as 1813 when Le Gallios first performed the task by squeezing rubberized pumping chambers between pairs of wooden planks [13]. But it was not until the 1960s when cardiac assist devices finally began to replace cardiopulmonary bypass circuits as a means to support the failing heart [14]. The earliest mechanical assist devices were pneumatically driven. The first implantable artificial ventricles in clinical use was reported by Liotta in 1963 and it consisted of a pneumatically-compressed valved tubular conduit that connected the left atrium to the descending aorta [14]. A double-lined restraint cup that wrapped around the ventricles and alternately inflated and deflated to displace blood from both ventricles (reported by G. Anstadt and P. Schiff in 1966) was also a pneumatic device [15]. An air-powered balloon pump that provides effective left ventricular unloading and systemic circulatory support by displacing blood from the descending aorta during the diastolic phase of the cardiac cycle was first used clinically in 1968 [16,17]. Around the same time, the idea of complete replacement of the entire heart using a pneumatic total artificial heart (TAH) emerged and the implantation procedure was first performed clinically in 1969 [18,19]. However, because these early attempts risked a high rate of fatality from sudden device failures, focus shifted toward the use of simpler single-chambered mechanical blood pumps for univentricular support, known as ventricular assist devices or VADs [3,18]. 

### 2.2. First Generation: Pulsatile Pumps

**Figure 1:**
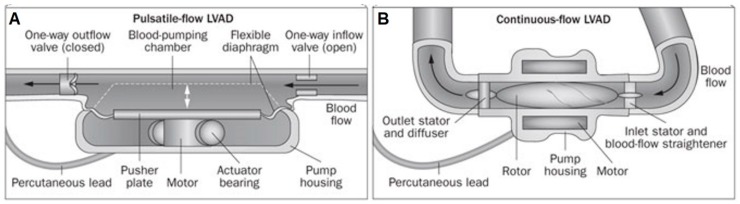
The first generation pulsatile-flow pumps (**A**) replicated the native cardiac cycle using a diaphragm and unidirectional artificial heart valves, while the second generation continuous-flow pumps (**B**) integrated a valveless axial pump designed to rapidly spin a single impeller.

When VADs were first developed, they were designed to replicate the native cardiac cycle and generate pulsatile flow using a diaphragm and unidirectional artificial heart valves (Figure 1A) [3]. The first generation VADs were either pneumatically or electrically driven and included larger pulsatile VADs like HeartMate XVE (Thoratec, Pleasanton, CA, USA) and Berlin Heart EXCOR (Berlin Heart, Berlin, Germany) that were used to support patients awaiting cardiac transplantation [18,20,21]. These earlier pulsatile pumps were characterized by their large size, heavy weight, and an external driving unit that seriously limited patients mobility. These first generation pulsatile VADs could be used either as a left ventricular assist device (LVAD), a right ventricular assist device (RVAD), or as a biventricular assist device (BiVAD).

#### 2.2.1. LVAD

Because the left ventricle (LV) and right ventricle (RV) can be supported either separately or in unison, ventricular assistance is commonly separated into LVAD, RVAD, and BiVAD categories [22]. With isolated LVAD therapy, the systemic circulation is typically supported by drawing blood from the left ventricular apex and pumping it into the ascending aorta. This not only restores perfusion to all organs and tissues outside the pulmonary circulation (including the heart itself), but also unloads the LV, which may prevent or even reverse pathologic LV remodeling caused by chronic pressure overload. Subsequent effects on RV function are complex however, as right-side improvements resulting from lower pulmonary pressures are offset by several factors that could lead to RV failure, including: increased preload, leftward shift and reduced contractility of the interventricular septum, increased work demand to match LVAD output, and tricuspid valvular distortions. The first successful LVAD implantation was completed by De Bakey in 1966, and the majority of cardiac support research has been dominated by LVAD developments for clinical practice ever since. Some first generation pulsatile LVADs include Novacor LVAS (Baxter Healthcare, Oakland, CA, USA), HeartMate I (Thoratec), and Thoratec PVAD (Thoratec) [18,23,24,25,26]. 

#### 2.2.2. RVAD

The clinical settings in which RVAD therapy are most commonly employed include acute myocardial infarction, pulmonary embolism, pulmonary hypertension, myocarditis, post-cardiotomy shock, cardiac transplantation, and LVAD implantation. As the frequency of LVAD use continues to rise, this last scenario is becoming increasingly common as nearly half of all CHF patients show right heart failure after LVAD implantation and 4% require RV support within the first two weeks post-operation [27,28]. Because RV complications after LVAD surgery are both relatively frequent and highly significant in terms of morbidity and mortality, the means to provide right ventricular mechanical support is now considered an essential capability in medical centers where LVAD therapy is performed [18,28]. Today, some RVADs like SynCardia (SynCardia Systems, Tucson, AZ, USA) serve as BTT while some others like Impella RP (AbioMed, Danvers, MA, USA), TandemHeart (CardiacAssist, Pittsburgh, PA, USA), and CentriMag RVAD (Thoratec) serve as peri-operative bridges to mechanical support [17,29]. 

#### 2.2.3. BiVAD

While the majority of patients retain sufficient RV function throughout the course of LVAD therapy to avoid the need for ancillary support, nearly 48% of LVAD recipients experience sufficient levels of postoperative RV dysfunction to warrant the use of a biventricular assist device [27]. BiVAD is especially helpful for patients with total heart failure because it supports both sides of the failing heart by balancing left and right pump flows and, in rare cases, inducing myocardial recovery. The first generation pulsatile BiVADs have saved many lives, but are limited by their bulkiness, the necessity of a large external pneumatic driver that inhibits patient mobility, infection at the driveline site, and thrombus formation. Some first generation BiVADs include AbioMed BVS5000 (AbioMed), Berlin Heart EXCOR (Berlin Heart), and Medos HIA-VAD (Stolberg, Germany) [27,30]. 

#### 2.2.4. Total Artificial Heart

Total artificial hearts (TAHs) are designed to entirely replace native heart function over extended periods to treat end-stage CHF. The first human TAH implantation was performed in 1969 by Denton Cooley using the Liotta artificial heart as a bridge to cardiac transplantation. The patient was supported on this pneumatic device for three days during which time hemolysis and deteriorating renal function prompted surgeons to replace the pump with a donor heart that failed 36 h later [18,19]. It was not until 1982 when the Jarvik-7 TAH (Jarvik Heart, New York, NY, USA) was able to support a patient for 112 days that these devices were generally considered a viable means to support patients for BTT [19]. CardioWest (SynCardia), which the Jarvik 7 later became, and Abiocor (AbioMed) are examples of TAHs that have been used clinically [31]. 

### 2.3. Second Generation: Continuous Axial Flow Pumps

Because first generation pulsatile pumps were limited by their large size, high noise emission, and durability issues leading to frequent malfunction and morbidity, research to develop smaller and more reliable devices were initiated and continued through the 1990s [17]. As a result of this work, Thoratec introduced a new VAD in 2001 called HeartMate II that was just one-seventh the size and one-quarter the weight of the original HeartMate XVE [20,21]. This radical design change was achieved by integrating a valveless axial pump with a variable magnetic field designed to rapidly spin a single impeller that produces continuous outflow directed in parallel to the axis of rotation (Figure 1B) [3]. HeartMate II received FDA approval for BTT in 2008 and for destination therapy in 2010 [32]. To date, over 26,600 patients have received HeartMate II LVAD demonstrating 85% survival at one year [33]. Other axial flow pumps developed during this same time period included Hemopump (Medtronic), DeBakey VAD (Micromed), HeartAssist-5 (Reliant Heart, Houston, TX, USA), Jarvik 2000 (Jarvik Heart), Impella (Abiomed), and Incor (Berlin Heart). These second generation LVADs were able to provide patients with a better quality of life, mobility, and restoration of heart function compared to the first generation positive displacement VADs, but still relied on extracorporeal power sources and required patients to undergo constant anticoagulation therapy for the duration of the implant due to the risk of thromboembolic events [18]. 

### 2.4. Third Generation: Continuous Centrifugal Pumps

**Figure 2:**
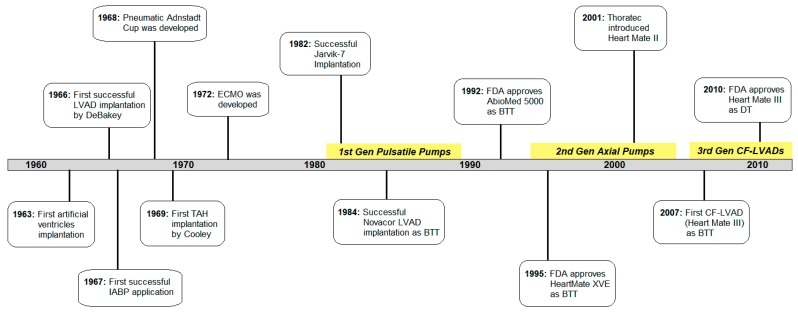
Timeline of important milestones of cardiac assist device (CAD) development history [38,39].

**Figure 3:**
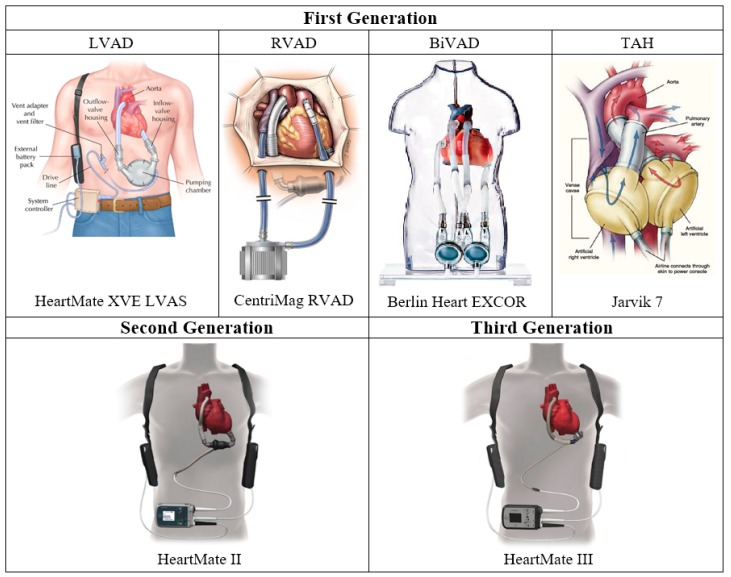
Examples of first, second, and third generation cardiac assist devices [40,41,42,43,44,45,46].

The third generation LVADs are continuous flow centrifugal pumps designed with magnetic and/or hydrodynamic levitation of the impeller with non-contact bearings and its outflow directed perpendicular to the axis of rotation [3,34]. These radial rotary pumps feature further reduced device size, noise emission, infection rate, and prothrombotic sites for better patient outcomes and lifestyles [18]. Now that nearly 99% of LVADs placed are continuous flow LVADs (CF-LVADs) today, third generation centrifugal pumps such as HeartWare HVAD (HeartWare), HeartMate III (Thoratec), CentriMag (Thoratec), Incor (Berlin Heart), Levacor (World Heart, Salk Lake City, UT, USA), and DuraHeart (Terumo Heart, Ann Arbor, MI, USA) play big roles [3,26]. HeartWare HVAD and HeartMate III received FDA approval for long-term mechanical circulatory support in 2017 and 2018, respectively, and CentriMag was approved to support one or both sides of the heart for up to 30 days in patients [35,36,37]. Some other milestones in VAD development history are summarized in the timeline shown in Figure 2, while some of the most popularly used first-, second-, and third-generation VADs are illustrated in Figure 3. Despite significant improvements in device function and durability, however, complications like right heart failure, infection, thrombosis, hemolysis, and neurologic events still persist [16]. 

## 3. Current State of The Art

### 3.1. CADs in Clinical Settings Today

After five-plus decades of dedicated research aimed at developing blood pump technologies to support the failing heart, a cadre of devices capable of delivering different levels of support at different levels of invasiveness are now available to treat different varieties and severities of cardiac malfunction. These range from acute catheter-based interventions used for partial univentricular support to long-term implantable pumps designed to restore normal perfusion levels in both systemic and pulmonary circulations [3,18,20]. Although guidance on patient selection for mechanical support is limited, the criteria usually include a combination of factors such as patient age, body size, cardiac malfunction type, disease stage, and candidacy for organ transplantation. For example, patients who require immediate VAD replacement due to the severity of their symptoms and/or are expected to have longer than normal wait times on the transplant list due to their body size and blood type are generally considered to be candidates for BTT devices. Alternatively, patients who require circulatory support but for some reason cannot be—or do not wish to be—listed for cardiac transplantation surgery are treated as destination therapy candidates [47,48]. 

#### 3.1.1. Short-term Circulatory Support

**Figure 4:**
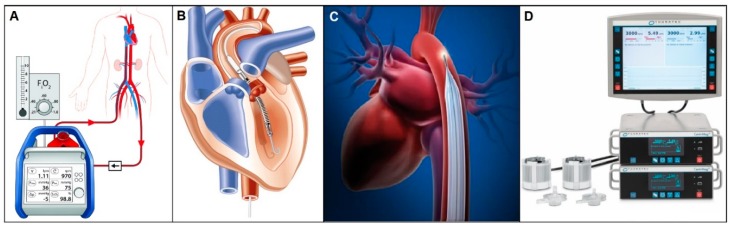
ECMO (**A**), AbioMed Impella (**B**), Teleflex Arrow IABP (**C**), and Thoratec CentriMag (**D**) are examples of temporary support mechanisms commonly used in clinical settings today [64,65,66,67].

Extracorporeal membrane oxygenation (ECMO) (Figure 4A) is a form of cardiopulmonary bypass that is used as a bridge to recovery, transplantation, or mechanical circulatory support [49]. It provides blood oxygenation and circulation with a mechanical pump stationed outside the body. ECMO is generally used in an emergent setting and continued until symptoms are improved, but the typical time course is hours to days because long-term ECMO support increases the likelihood of thrombotic complications [49,50]. Even though ECMO has been in clinical use as a class II/III device for over 30 years, the decision to use it remains a risk vs. benefit situation because complication rates are high as occurrences of bleeding and infection reach up to 40% and 31%, respectively. Patients with neurologic injuries, hemorrhage, immunosuppression and/or advanced age are generally thought be poor candidates for ECMO treatment [51,52]. 

AbioMed’s Impella catheter (Figure 4B) is an intravascular microaxial blood pump that provides partial circulatory support from a few hours to one month maximum [53,54]. Left ventricular Impella catheters come in three different models: Impella 2.5, Impella CP, and Impella 5.0, which produce flow rates up to 2.5 L/min, 3.5 L/min, and 5.0 L/min, respectively. All three are designed to circulate blood by placing their inlet in the LV and outlet in the ascending aorta. Similarly, there is Impella RP designed for partial right sided circulatory support, which provides up to 4.0 L/min of blood flow to the pulmonary circulation. Just last year in 2018, Impella Ventricular Support System received approval for expanded FDA indications for cardiomyopathy and percutaneous coronary intervention procedures after demonstrating its safety and effectiveness on over 50,000 patients treated from 2008 to 2017 [55,56,57]. One major caveat with these devices is that their proper function is highly dependent on the correct position of the catheters, which makes post-implant management of these catheter-based pumps critically important. All models come with an Automated Impella Controller (AIC) that monitors and controls the overall system [58]. 

Pneumatic intra-aortic balloon pumps (IABP) (Figure 4C), which are internal counterpulsation devices placed inside the descending aorta, has been one of the most common mechanical support systems for the failing heart ever since it was classified as a class III device in 1979 [59,60]. The balloon is inflated during ventricular diastole to increase diastolic pressure, coronary blood flow, and systemic perfusion, and rapidly deflated during systole to induce reduced cardiac afterload and enhanced cardiac output [61]. The IABP is actually one of the earliest CADs developed, with the first preliminary studies done as early as 1961 by Kanitrowitx and Moulopoulos and the first successful clinical application reported in 1967 [59,61]. Most IABPs in clinical use today are predominantly Arrow IABP series, now acquired by Teleflex Medical. Because proper actuation timing is crucial for counterpulsation therapy, Teleflex Arrow IAB Catheters come with their own AutoCAT2 control unit that has both AutoPilot Mode, which automatically selects appropriate settings using arterial pressure waveforms as the guideline, and Operator Mode in which all settings are user-controlled. The catheter balloons also come in different sizes for different sized patients [61]. 

Thoractec’s CentriMag acute circulatory support system (Figure 4D), a temporary external VAD that can support right, left, or both ventricles, was the first and only magnetically levitated blood pump cleared by FDA in 2008 [62]. It is a continuous flow centrifugal pump without bearings or seals that operates at speeds up to 5500 rpm, delivering up to 9.9 L/min blood flow for a maximum recommended support duration of 6 h [63]. This short-term solution for acute heart failure features a magnetically-levitated pump impeller that operates within a contact-free environment to help minimize blood-related complications. The CentriMag system comes with a pump, a motor, a console with dual display monitor, a back-up console battery with a 5-hour recharge time, and a power conditioning unit that is air transport operable with AC power and able to accommodate up to four CentriMag consoles simultaneously [63]. 

#### 3.1.2. CADs for Extended Use

Although the Heartmate II axial flow pump remains the world’s most widely used and extensively studied VAD to date with over 26,000 patients implanted for periods up to 10 years and beyond, third-generation centrifugal pumps like the HeartMate III (Thoratec) and HeartWare HVAD (HeartWare) are currently poised to become the device of choice as either BTT or DT for end-stage CHF patients. HeartMate III (Figure 4) was built upon the HeartMate II platform but with key improvements that include a modular driveline, mobile power unit interface, no surgical pocket, less power consumption, and most importantly, a unique magnetically levitated core system called Full MagLev technology [68]. This proprietary maglev system reduces overall blood trauma and maximizes hemocompatibility by maintaining large and consistent gaps within the pump housing and features an optional pulse mode as a means to minimize stasis and provide pulsatile flow to perfused organs. This design allows for significantly less shear stress (hemolysis) and blood-contacting surface area (thrombosis) since the size of the flow path that allows red blood cells to pass without rotor-housing contact is more than 20 times larger than that of its predecessor [68]. In addition to the pump itself, the HeartMate III system comes equipped with an external controller that powers and checks the pump and driveline, a percutaneous driveline, and an external battery pack. Refined implantation techniques together with improvements in mechanical reliability, pumping efficiency, and battery life have increased 2-year survival rates from 76.2% to 82.8% while also contributing to surgical ease and patient quality-of-life [69,70]. 

HeartWare HVAD is a small CF-LVAD with a displacement volume of 50 mL and an output capacity of 10 L/min [71]. It is characterized by a unique wide-blade impeller and a hybrid magnetic-hydrodynamic suspension technology that ensure no mechanical contact within the pump and a dual-motor system that is designed for increased efficiency and reliability [34]. It comes with a rotary pump that operates at speeds ranging from 1800 to 4000 rpm, a percutaneous driveline, an external microprocessor-based controller, a monitor that displays and logs downloadable waveform data, lithium-ion batteries that allows patient mobility for about 4 to 6 h, AC/DC power adapters, and a battery charger. Its small device size and cannula allow minimal invasiveness and therefore faster postoperative recovery and better clinical outcomes [34,71]. 

SynCardia CardioWest TAH (SynCardia) is the world’s first and only commercially approved total artificial heart that is currently in use today [72]. The mechanics of the device are fairly simple. It delivers pulsatile flow up to 9 L/min by filling two artificial ventricles that are sutured to the patient’s aorta and pulmonary artery and ejects blood through unidirectional valves via a pneumatically driven diaphragm [31]. According to INTERMACS reports, SynCardia TAH recipients experienced significantly fewer neurologic and thromboembolic events compared to BiVAD recipients [31]. It has notably increased patients support time and currently has an overall one-year survival rate of 67.6% [73].

#### 3.1.3. Pediatric Pumps

Conventional continuous flow VADs were designed specifically to treat adult patients, who comprise the vast majority of the end-stage CHF population and so tend to be too large for use in pediatric patients weighing less than 25 kg (55 lbs.) [74]. Berlin Heart EXCOR Pediatric is a pulsatile paracorporeal VAD designed for left and/or right ventricular support of young patients from newborns to adolescents [75]. It is composed of a cannula that comes in different tip types and sizes, a blood pump that also varies in sizes from 10 to 60 cc, and a driving unit that provides alternating pneumatic pressures. The system can be powered by either the stationary IKUS driving unit or a portable battery unit that lasts for roughly 6 h. To monitor patients, the IKUS unit is integrated with laptop software that is programmed to log and store data as well as alarm both visually and audibly when waveform readings are abnormal [75]. Besides Berlin Heart EXCOR, other pediatric pumps or miniature adult pumps include the Jarvik 2000 (Jarvik Heart) that come in different sizes for children and infants, PediaFlow (PediaFlow Consortium, Pittsburgh, PA, USA) that supports infants and young children weighing 2–25 kg, the miniature MVAD HeartWare (HeartWare), and CircuLite (CircuLite Inc., Saddle Brook, NJ, USA) [76]. 

### 3.2. Clinical Complications of Current VADs

In spite of the increasing number of VAD options currently available to patients due to revolutionary advances in cardiac support technologies, numerous challenges still persist. Ventricular arrhythmia, right heart failure, infection, pump thrombosis, and bleeding are still areas of concern, as are issues of long-term patient management and a lack of clear guidelines regarding patient eligibility criteria for VAD therapy [77]. Difficulties in gauging the likelihood of therapeutic benefit for any given individual HF patient is thought to be the biggest reason behind the recent plateauing of VAD use. Optimization of the treatment process and refinements in patient selection criteria are therefore needed to promote further improvements in survival rate and patient quality of life, especially in the setting of long-term circulatory support.

Indeed, given that heart failure has now risen to pandemic proportions across the globe while the availability of donor hearts remains woefully inadequate to meet the rising demand, continued expansion of mechanical circulatory support for use as long-term BTT or DT is considered a clinical necessity. But despite decades of development most VAD therapies are limited to short-term BTT applications due to three longstanding complications. One is bacterial infection from percutaneous drivelines, which is the most frequent LVAD-associated problem [5]. Another is thromboembolic events associated with blood-contacting surfaces, which includes both pump thrombus formation and blood clotting in the circulatory system [3,6]. And the third is bleeding, mainly at the surgical site during the early postoperative period and gastrointestinal bleeding that usually begins three months after continuous flow LVAD implantation [78]. 

#### 3.2.1. Driveline Infections

**Figure 5:**
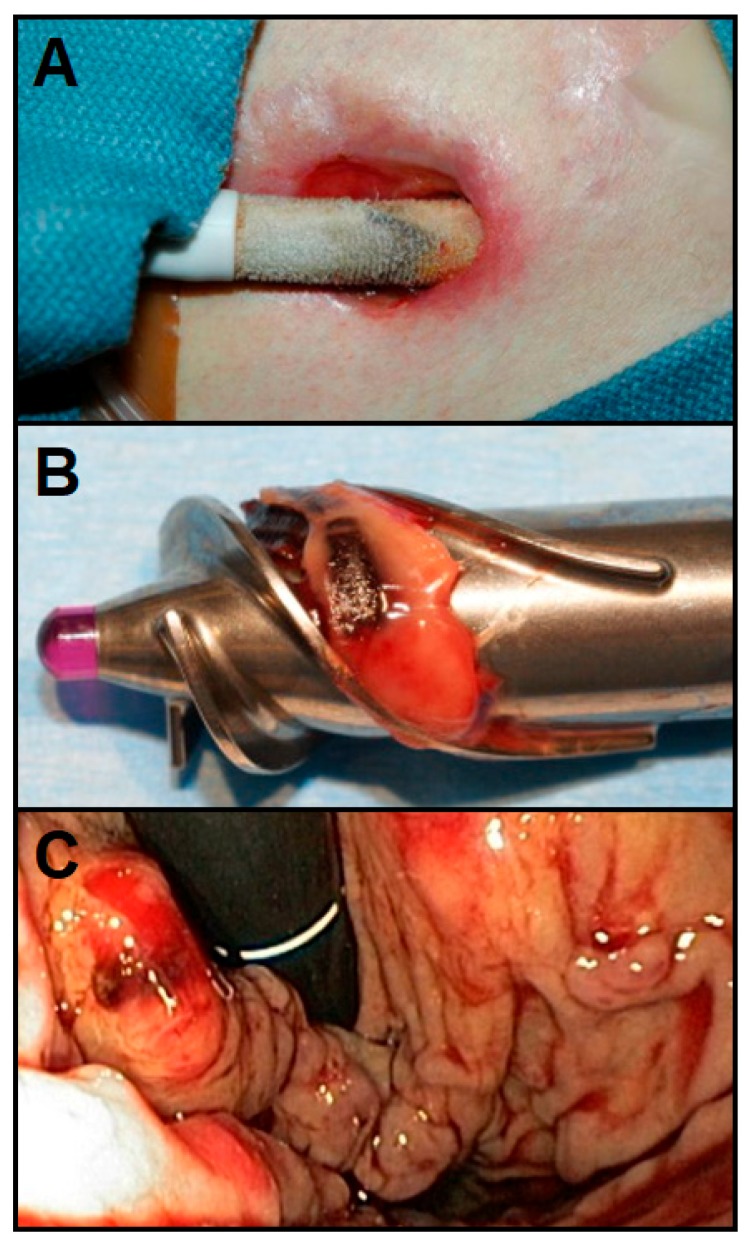
Some of the most longstanding complications after left ventricular assist device (LVAD) implantations are driveline infections (**A**), pump thrombosis (**B**), and gastrointestinal bleeding (**C**) [83,84,85].

Device malfunction, bleeding, thrombosis, and inadequate aftercare all contribute to VAD failure in the clinical setting, but percutaneous driveline infection (DLI) (Figure 5A) is one of the most common cause of mortality with these devices, accounting for 47% of all unplanned readmissions for LVAD patients [18,30]. This risk factor has proven difficult to avoid in these pumps as drivelines that provide power, control, and communication are percutaneously sutured to remain secure, and this driveline exit site creates a conduit for bacterial entry that often leads to DLI. The prevalence and seriousness of DLI, which often leads to erythema, hyperthermia, purulent drainage, and significantly lower survival rate, increased as LVAD therapy expanded from short-term to long-term use [3,16]. Although approximately 70% of infected patients require rehospitalization in the first year, there currently is no comprehensive guideline for DLI treatment besides general precautions like minimal exit-site movement, long-term suppressive antibiotics, and antimicrobial therapy [20,21,79,80]. Ongoing efforts to decrease DLI incidents include optimization of driveline implantation techniques and minimization of pump profile and operational invasiveness, which has resulted in smaller and more efficient devices such as the entirely intra-pericardial HVAD (HeartWare) and completely intra-thoracic HeartMate III (Thoratec) [16]. However, in all cases a tunneled percutaneous driveline is still required for power delivery from sources outside the body [17]. 

#### 3.2.2. Pump Thrombosis

Another significant cause of LVAD complications is thromboembolism (Figure 5B) associated with blood-contacting surfaces [3,6]. Pump thrombosis, where blood clots form at the blood-device interface, is a multifactorial process caused by misuse of anticoagulants, abnormal angulation of cannulas, and surface mediation of blood-contacting devices [3]. Thrombosis can occur in any component of the LVAD in contact with the bloodstream and may result in turbulent flow, elevation in device power consumption and, in extreme cases, inability to unload the LV [21]. The annual incidence of pump thrombosis in LVAD patients exceeds 10%, of which nearly one third lead to serious complications including aortic insufficiency, hemolysis, neurologic events, and cardiogenic shock [20,21]. From the time of confirmed pump thrombosis, there is a two-fold increase in mortality at 30, 90, and 180 days, where mortality reaches 48.2% if no LVAD exchange or cardiac transplantation is performed within that given time [20,21]. This potential complication, common to all blood-contacting devices, requires VAD recipients to undergo costly—and potentially dangerous—anticoagulation therapy for the duration of the implant period. In order to minimize the rate of chronic pump thrombosis, innumerable changes in VAD designs have been made over the years. Modern LVAD surface area has been scaled down, impeller profiles have been adjusted, implantation invasiveness has been minimized, and less reactive surface materials have been chosen. Nonetheless, the risk persists and long-term antithrombotic therapies including anticoagulant drugs, antiplatelet agents, and routine surveillance are still required by patients receiving VAD therapy [20,21]. 

#### 3.2.3. Gastrointestinal Bleeding

The reported incidence of gastrointestinal bleeding (GIB) (Figure 5C) after continuous flow LVAD implantation is alarmingly high, as much as 61% by one account [81,82]. There are several factors leading to GIB syndrome, but with third-generation continuous flow pumps the low pulsatility flow profile combined with increased oxidative and shear stresses seem to cause hematological abnormalities such as platelet dysfunction and von Willebrand factor (vWF) degradation [21,81]. And chronic anticoagulative treatments like warfarin and antiplatelet agents like aspirin administered to prevent clot formation at blood contacting surfaces only worsen the risk of bleeding [21]. GIB can be initially diagnosed and evaluated with endoscopy, but the most appropriate method of treatment after diagnosis is not always clear due to difficulties identifying the causes that underlie this complicated syndrome. Currently, a multidisciplinary approach that considers the location and severity of bleeding and thrombosis simultaneously is being used to manage GIB, but better insights into the etiology and treatment of GIB are still being sought to improve outcomes [81,82]. 

## 4. Innovations for Effective Long-Term Cardiac Support

The rate of DLI, thromboembolic incidents, and bleeding problems must be curtailed if long-term cardiac support is to become a viable treatment option for end-stage CHF patients. Toward that end, there have been numerous attempts to eliminate these predominant failure modes and develop an untethered, non-blood-contacting VAD as a destination therapy.

### 4.1. Alternative Powering Methods for Untethered Cardiac Support

To provide long-term CAD patients better quality-of-life, various powering methods have been proposed to minimize or eliminate extracorporeal power requirements that limit patient autonomy and contribute to patient stress over potential power delivery failures (e.g., driveline fracture and battery exhaustion) and DLI risk. One of the most interesting attempts was a nuclear-powered device from the 1980s that used Plutonium-238 as a power source. The potential was in the nuclear radioisotope Plutonium that offered the highest possible energy density and long half-life without requiring any energy storage. However, the critical problems of heat dissipation and safety concerns regarding nuclear element leakage eventually led to termination of the project [86,87]. 

Another attempt to develop a permanently implanted circulatory support system was based on a small, lightweight spring decoupled C-core solenoid that was first introduced in the early 1980s. This solenoid drive system was used to actuate a pair of preloaded beam springs that directly coupled to a dual pusher-plate blood pump, producing high starting forces and constant pump pressures through repeated ejection strokes. With this technology operating in combination with external plug-ins that included portable and rechargeable lithium-ion, nickel metal hybrid, or lead acid gel batteries, a level of patient mobility similar to that afforded by battery-powered devices available today was achieved [88,89]. However, this drive system was limited not only by requiring patients to carry around external hardware like charging stations, battery packs, and emergency back-up systems in a backpack, but also by the anxiety produced by having to charge batteries every few hours [88,89]. When findings from device malfunction cases were reviewed, researchers found that there were significant numbers of hospital visits due to device alarm of unknown origin and/or actual malfunctions resulting in controller exchange or battery change. Although not all cases represented serious clinical complications, device alarms and malfunction notices caused severe levels of anxiety and considerably reduced patient quality of life [90]. Much worse, in some cases, patients actually died from battery exhaustion because of unexpected events that drained the batteries before they could be recharged [91]. Therefore, alternative power sources for untethered pump operation have been sought to create totally implantable devices that are safe, reliable, and relatively maintenance-free.

#### 4.1.1. Transcutaneous Energy Transfer System

**Figure 6:**
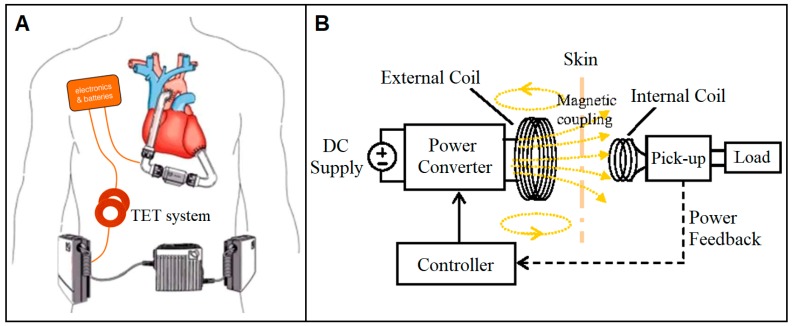
Schematics of the TET system (**A**) in patient use and (**B**) with an electromagnetic coupling between the internal and external coils located inside and outside of patient skin, respectively [100,101].

Transcutaneous energy transmission (TET) technology (Figure 6A) that transfers power across intact skin makes devices completely implantable and therefore free of the risk of DLI [17]. At a time when over 20 million Americans are estimated to have some type of implanted medical device, the TET system sounds extremely appealing [92]. The idea of an inductive coupling of two coils that transfers electromagnetic energy at radio frequencies across a closed chest wall was first described by Schuder and colleagues in 1961 [93]. Because VADs tend to demand a higher range of power (up to about 25 W) compared to other implants like pacemakers or implantable cardiac defibrillators, transmission efficiency and the total amount of transferrable power are key performance criteria. Different methods of transmitting energy across skin such as ultrasonic energy transfer and acoustic energy transfer have been previously developed, but because inductive coupling TET outperforms the others by more than double in terms of efficiency, the latter has been used in devices like AbioCor TAH (AbioMed), which was FDA approved as a permanent TAH for humanitarian uses in 2006, and the LionHeart LVAD (Arrow International, Reading, PA, USA), which received FDA approval for Phase I human clinical trials in 2001 [93,94,95,96,97,98]. 

The inductive electromagnetic TET system (Figure 6B) used in these devices has proven to be a promising wireless powering method that sufficiently meets the power transmission requirement of up to 25 W [92,99]. When studied with 14 AbioCor TAH patients, 30-day survival rate was 71% with no device-related infections reported, which clearly demonstrated the value of the TET system regarding the elimination of DLI risks [93]. However, this tether-free system is significantly limited by its power transmission range since the transmit and receive coils must remain very close together (within a few millimeters). This proximity restriction requires the receive coil to be implanted just under the skin and the external transmit coil to be secured in a single position on the skin surface with an adhesive dressing [93]. The two coils can be distant for a very brief period of time (about 30 min), allowing activities like a brief shower [96]. Another limitation is its lower energy efficiency compared to conventional extracorporeal drivelines as the TET system consumes approximately 20% of the generated power during operation [93]. Other drawbacks like fatal component failure, bleeding, and pain due to the large cumulative volume of all implanted parts also play a big role in preventing TET technology from being the main VAD powering method today [3]. 

#### 4.1.2. Muscle-powered VADs

The use of electrically stimulated skeletal muscle as an endogenous power source to drive circulatory support systems is another alternative that is currently under study. An internal muscle energy converter that operates by converting the contractile energy of a muscle into a hydraulic power source would greatly simplify cardiac implants by eliminating electromechanical components and avoiding the need to transmit energy across the skin [102,103]. A device powered by contractile energy and controlled via a pacemaker-like device implanted beneath the skin could, in principle, provide a safe, tether-free means to support the failing heart over extended periods of time. 

The concept of muscle-powered cardiac support is not new. The use of untrained skeletal muscle to aid the failing heart dates way back. In 1935, Beck and Tichy employed static muscle grafts to revascularize the myocardium [104]. And in 1958, Kantrowitz isolated diaphragm muscles in dogs to form pouches for use as ‘myocardial substitutes’ [104]. But in 1969 the concept of muscle-powered cardiac assist was given new life when Salmons and Jarvis demonstrated that myofiber properties can be changed from glycolytic fast type to oxidative slow-phenotype via muscle impulse activity training [104]. This key discovery opened a whole new realm of possibilities involving conditioning skeletal muscle to provide fatigue-resistant long-term circulatory support.

**Figure 7:**
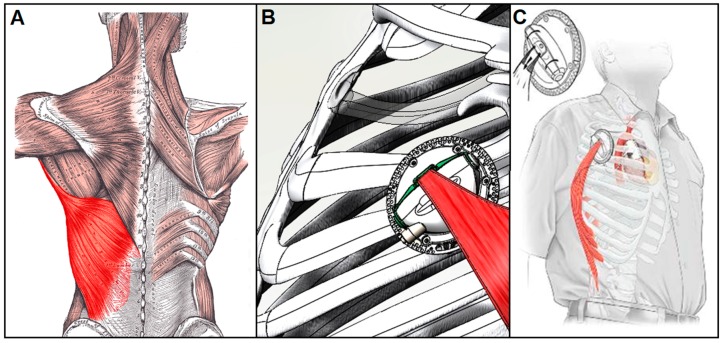
Muscle-powered VADs could use the latissimus dorsi (**A**) as its power source and convert this endogenous muscular power into hydraulic energy via a completely implantable muscle energy converter (**B**) that can potentially power pulsatile VADs for long-term use (**C**) [103,106,107].

The recent development of a functional muscle energy converter (MEC), which operates by converting endogenous muscle energy into hydraulic power, may ultimately provide CAD developers with the means to harness the body’s own energy to assist the failing heart over the long term [102,103]. Among the several large skeletal muscles that might conceivably be used for this purpose, the MEC targets the latissimus dorsi muscle (LDM) (Figure 7A) due to its large size, surgical accessibility, proximity to the thoracic cavity, and steady-state work capacity sufficient for long-term cardiac support [105]. Trained LDM controlled by a programmable pacemaker-like cardiomyostimulator that coordinates muscle activity with the cardiac cycle has been shown to produce mechanical power at levels sufficient for pulsatile VAD actuation [103,104]. As the current MEC (Figure 7B) has been optimized to operate at contractile force and velocity levels that correspond to peak power generation in fully-conditioned human adult LDM, its potential as a means to power a completely self-contained VAD (Figure 7C) for long-term use is promising [102]. 

### 4.2. Non-Blood-Contacting Cardiac Assist Devices

Despite innumerable CAD designs and material modifications made over the decades in an attempt to eliminate chronic pump thrombosis, the situation still persists while the precise dosage and frequency of long-term antithrombotic therapies remain ambiguous [20,21]. Consequently, several groups are currently working to avoid this problem by designing non-blood contacting devices [3]. These devices are intrinsically pulsatile and can be programmed to deliver energy to the bloodstream during cardiac systole (*copulsation*) or diastole (*counterpulsation*). Copulsation enhances cardiac output by increasing pulse and arterial pressure during systole, while counterpulsation boosts heart function by reducing aortic pressures as the heart fills thereby providing lower cardiac afterload for the failing heart [3]. These techniques have been shown to significantly increase aortic peak pressure, cardiac output, and regional and coronary blood flow [108]. But, above all, the most critical advantage these technologies offers is that they can be applied without touching the blood stream. 

#### 4.2.1. Copulsation Direct Cardiac Compression Sleeve

A normal heart with a ventricular ejection volume of about 71.5 mL per beat (CO = 5 L/min and HR = 70 bpm) has a ventricular ejection fraction (EF) of 60%. While a healthy heart’s EF ranges from 55% to 70%, anything less than that is considered mild (<54%) to severe (<35%) heart failure. One way to boost the EF of a defective heart is by applying pulsatile pressure to the epicardial surface in synchrony with the natural ventricular contraction. 

Copulsative biventricular compression devices have been around for decades. The Anstadt Assistor Cup became the first successful direct cardiac compression sleeve (DCCS) in 1991 and Dr. DeBakey’s pneumatic LV compression cup was first implanted in 1996 [20,21,109]. As these preliminary ventricular DCCSs showed successful increases in arterial pressure and cardiac output, more pneumatic and electric sleeves were developed including the “cuff-like” Heart Booster (AbioMed) that covers and compresses the heart with parallel compression tubes [110], Mannequin (Chase Medical, Richardson, TX, USA) that restores round-shaped ventricles to its original oval-shape [111], and Heart Blanket (Leeds University, UK) that gives underperforming hearts an extra boost by contracting ventricles with piezoelectric bands in synchrony with pacemaker stimulations [112]. 

**Figure 8:**
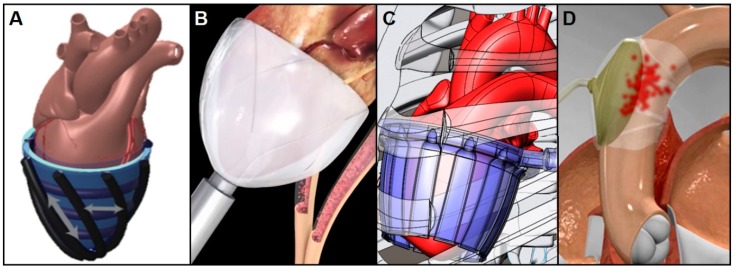
Biomimetic (**A**), minimally invasive (**B**), and muscle-powered (**C**) soft robotic direct cardiac compressive sleeves (DCCS) use copulsation and extra-aortic balloon pumps (EABP) (**D**) use counterpulsation techniques to enhance cardiac function without directly interacting with the bloodstream [107,108,113,114,117].

Recently, researchers have turned to emerging soft robotic technologies to improve the long-term functionality of DCCSs. In 2017 for example, a silicone molded sleeve (Figure 8A) that employs McKibben pneumatic artificial muscles (PAMs) placed helically and circumferentially to both compress and twist the heart without contacting blood gathered a lot of attention [113]. This soft robotic sleeve made of two biomimetic layers of contractile elements that shorten when pressurized during ventricular systole was able to restore cardiac output to 88% of normal when tested on porcine hearts [113]. CorInnova’s minimally invasive soft robotic DCCS (Figure 8B) is a collapsible self-deploying device that wraps around the ventricles with custom fit thin-filmed pneumatic chambers. They were able to increase cardiac output by up to 50% in large animal acute heart failure studies [114]. Unlike these pneumatic devices that are tethered to an external air supply, a muscle-powered DCCS (Figure 8C) that uses the geometric advantage produced by an array of thin-walled tubes is currently under development [107]. This sleeve comprises hydraulically driven tubing arrays that contract and expand circumferentially when filled and emptied. As fluid enters the array of thin-walled polymer tubes connected side-to-side it transforms each tube from a flat (deflated) to a circular (inflated) cross-section to effectively compress the epicardial surface in synchrony with ventricular ejection, ultimately leading to enclosed ventricular blood volume changes as high as 60% [107]. This hydraulic DCCS device combined with the MEC technology introduced above could, in principle, allow for the development of a completely untethered, muscle-powered, non-blood-contacting VAD for long-term cardiac support.

#### 4.2.2. Counterpulsation Extra-Aortic Balloon Pump

Another form of circulatory support for CHF patients that provides effective cardiac unloading and patient stabilization is displacement of blood from the aorta during the diastolic phase of the cardiac cycle. This technique is most often performed clinically using an IABP that is implanted and inflated inside of the descending aorta as previously described. This mechanical support augments diastolic pressure and coronary circulation via balloon inflation and reduces the resistance to systolic output via the presystolic deflation of the balloon [16,17,59,115]. The biggest factor that prevents this technology from becoming a viable method of long-term support is the fact that it is often associated with thromboembolism with extended use due to its direct interaction with the blood stream. Therefore, an extra-aortic balloon pump (EABP) (Figure 8D) that wraps and compresses the external surface of the ascending aorta like the C-pulse device (Sunshine Heart Inc., Eden Prairie, MN) may offer clinicians an alternative solution. The C-pulse counterpulsation EABP was clinically tested and shown to significantly increase aortic peak diastolic pressure, cardiac output, and regional and coronary blood flow without touching the blood [103,116]. In the context of long-term cardiac support it is worth mentioning that, like the soft robotic DCCS, this device also has the potential to be driven by muscle-powered actuation, which would allow for durations of use far beyond what is now possible with pneumatic actuation.

#### 4.2.3. Passive Periventricular Restraint

Passive periventricular restraint, which involves wrapping the entire epicardial surface with a sleeve-like prosthetic to provide circumferential diastolic support to the failing heart, is an approach that evolved from a surgical procedure known as cardiomyoplasty (CMP) in which the ventricles were wrapped with the latissimus dorsi (LD) muscle flap and stimulated to contract in synchrony with the systolic portion of the cardiac cycle. While CMP was effective in reducing wall stress, myocardial oxygen consumption and adverse ventricular remodeling, these benefits were found to persist in some patients even after the muscle flap stopped contracting, which suggested that these same effects might be produced via passive ventricular restraint alone. Toward that end, several passive prosthetic devices were developed to produce the same effects without resorting to the surgical complexities and post-surgical complications involved with LD flap isolation and subsequent transplantation into the chest. Corcap (Acorn Cardiovascular, Saint Paul, MN, USA) and Paracor HeartNet were two such devices that were designed to act more like the passive LD flap insofar as pressure was applied uniformly across the ventricular free walls. The Acorn sleeve was a flexible, polyethylene-terephthalate mesh that was placed around the heart through a median sternotomy to provide end-diastolic support and reduced wall stress [118]. The Paracor device was formed from Nitinol wire mesh encased in silicone that exerted continuous elastic force on the heart throughout the cardiac cycle and could be deployed over the ventricles via an introducer sheath positioned over the cardiac apex through a mini-thoracotomy [119]. Both devices were tested in limited clinical trials but, despite showing positive LV remodeling in a subset of patients with dilated cardiomyopathy, neither produced significant improvements in patient survival or quality of life and were subsequently taken off the market. 

### 4.3. CADs in Summary

Key characteristics of the large and expanding family of cardiac assist devices developed in the past, used in the present and slated for the future are summarized in Table 1 below.

### 4.4. Patient Management for Long-Term Treatment

Since 2001 when the Randomized Evaluation of Mechanical Assistance for the Treatment of Congestive Heart Failure (REMATCH) trial became the landmark study that established the benefits of implantable, pulsatile, and permanent VAD therapy in patients with late stage CHF, survival rates have improved to nearly 80% one-year after primary implantation due to a combination of refinements in patient selection strategy, surgical techniques, and peri-operative management [3,19,131,132]. Even though the survival rate has gone up, late stage CHF patients still suffer from physical and psychological distress stemming from the lack of mobility and freedom. As the 2018 ENDURANCE supplemental trial concluded, the ideal form of destination therapy should provide effective and comfortable long-term mechanical support with an emphasis not only on prolonging survival, but also reducing morbidity and improving overall quality-of-life [133]. Considering that there are currently no practice guidelines for patient management, there is an urgent need for a more systematic and organized protocols for these patients. As the PREVENT trial highlights, more seamless, real-time communication between patients and caregivers is needed [17]. Devices like CardioMEMS (St. Jude Medical, St. Paul, MN, USA) and HeartAssist-5 (ReliantHeart Inc., Houston, TX, USA) that have sensor and remote monitoring capabilities via cell phone or other portable devices were developed to meet this critical need [17,134]. Overall, VADs with long-term reliability and low complication rates in combination with proper postoperative and follow-on care will together establish what may be considered a true destination therapy.

## 5. Conclusions

Since its inception in the early 1960s, a remarkable amount of research and development has been performed in an effort to improve and expand the field of cardiac assist devices. As a result, a wide array of cardiac assist technologies is available to clinicians today, each with their unique set of strengths and weaknesses, but all designed with one common goal in mind: to provide safe, reliable circulatory support however and whenever it is needed. 

Of course, these challenges grow larger as rising levels and durations of support are required and it is important to continue to seek solutions that will free these patients from persistent physical risks and psychological distress. Toward that end, reducing device-related complications and eliminating the loss of freedom imposed by percutaneous tethers will be key factors in developing CADs that are truly suitable for long-term or permanent use. In addition, replacing current patient management practices with physician–patient interface systems that are more systematic, convenient, and effective will likely play a big role in improving the lives of CHF patients who must rely on life-sustaining devices for years on end. Fortunately, there is reason to expect that many of these improvements will be implemented in the not-too-distant future as steps to meet these challenges are currently being taken by several groups working to develop effective destination therapies with longer patient survival times and improved quality-of-life. 

## Figures and Tables

**Table 1:** Commonly used cardiac assist devices and their key characteristics (*IVC: inferior vena cava, FA: femoral artery, LA: left atrial, PA: pulmonary artery) [35,120,121,122,123,124,125,126,127,128,129,130].

Category	Product	Type of Support	Duration of Support	Advantages	Limitations
Early Methods of Cardiac Support	ECMO	BiVAD	Short-term	Extracorporeal artificial heart-lung bypass for acute support	Upper body hypoxia, LV dilatation, thrombosis
	IABP	Descending Aorta	Short-term	Increases myocardial oxygen perfusion and cardiac output	Thrombosis, aortic rupture, arterial flow obstruction
1st Generation—Pulsatile Flow	HeartMate XVE	LVAD	Long-term	Improved enough to receive FDA approval for DT in 2003 and CE mark in 2004	Bulky and Heavy
	Berlin Heart EXCOR	BiVAD	BTT	Pediatric uses with various pump sizes	Not completely implanted
	Novacor LVAS	LVAD	BTT	Longer durability and higher reliability at the time	Still large and bulky with three extracorporeal hardware
	HeartMate I	LVAD	BTT/BTR	Introduced textured blood contacting surface to reduce thrombosis	Large size and complications like bleeding and driveline infection
	Thoratec PVAD	Uni or BiVAD	Short-term	Weeks to months support for patient’s home discharge post-cardiotomy	Common side effects from pneumatic driveline
	ABioMed BVS 5000	Uni or BiVAD	Short-term	Resuscitate critically ill patients for acute stabilization	Risks of bleeding, coagulopathy, and end-organ damage
	Jarvik 7	TAH	Long-term	World’s first permanent total artificial heart; more used as a BTT now	Thrombotic deposition and cerebral embolic events
	AbioCor TAH	TAH	Long-term	Uses TET technology without aid of wires	Discomfort with TET system, bulkiness, clotting at device surfaces
	ABioMed Impella RP	IVC-to-PA	Short-term	First and only FDA approved percutaneous heart pump for RV support	Thrombotic vascular complications and hemolysis
	Tandem Heart	LA-to-FA	Short-term	Significantly reduces preload and augments cardiac output	Risks of cannula migration, thromboembolism, and cardiac tamponade
2nd Generation—Continuous Axial Flow	HeartMate II	LVAD	Long-term	FDA approval for DT, Improved survival rate and patient quality of life, Most commonly installed LVAD in 2000s	Bleeding, cardiac arrhythmia, infection, sepsis
	Heart Assist 5	LVAD	Long-term	Small size and weight, CE mark approved remote monitoring system in 2012	Bleeding, thrombosis, infections
	Jarvik 2000	LVAD	Long-term	Pediatric uses, FDA approval for trial using as a DT in 2012	Class 2 device recall for a potential external cable damage in 2018
	ABioMed Impella	FA-to-LV	Short-term	Minimally invasive, Varying sizes	Hemolysis, aortic valve injury, infection
3rd Generation—Continuous Centrifugal Flow	HeartWare HVAD	LVAD	Long-term	Small size, magnetically levitated rotor, FDA approval for DT in 2017	Risks of infection, bleeding, arrhythmia, stroke
	HeartMate III	LVAD	Long-term	Magnetically levitated rotor, FDA approval for DT in 2018	Risks of infection, bleeding, arrhythmia, stroke
	DuraHeart	LVAD	Long-term	Favorable clinical outcomes as BTT in Japan and Europe	Hemolysis, thromboembolism, bleeding
	HeartWare MVAD	LVAD	Long-term	Miniature size for pediatric uses	Risks of infection, bleeding, and thrombosis
	CentriMag	Uni-VAD	Short-term	Magnetically suspended rotor for acute therapy, Minimal shear force on RBCs and hemolysis	Bleeding, infection, respiratory failure, hemolysis, neurologic dysfunction
Non-blood-contacting VADs	CorInnova	Ventricular Epicardium	Potentially Long-term	Minimally invasive, Non-blood-contacting, soft material	Studies done on large animals only
	Biomimetic DCCS	Ventricular Epicardium	Potentially Long-term	Soft material, Non-blood-contacting, compression and torsion applications	Still under development
	Muscled-powered DCCS	Ventricular Epicardium	Potentially Long-term	Tether-free, Non-blood-contacting, Biocompatible soft material	Still under development
	C-pulse Device	Ascending Aorta	Short-term	Non-blood-contacting	No longer commercially available
